# Impact of Meditation-Based Lifestyle Practices on Mindfulness, Wellbeing, and Plasma Telomerase Levels: A Case-Control Study

**DOI:** 10.3389/fpsyg.2022.846085

**Published:** 2022-03-04

**Authors:** Nirodhi Namika Dasanayaka, Nirmala Dushyanthi Sirisena, Nilakshi Samaranayake

**Affiliations:** ^1^Research Promotion and Facilitation Centre, Faculty of Medicine, University of Colombo, Colombo, Sri Lanka; ^2^Department of Anatomy, Genetics and Biomedical Informatics, Faculty of Medicine, University of Colombo, Colombo, Sri Lanka; ^3^Department of Parasitology, Faculty of Medicine, University of Colombo, Colombo, Sri Lanka

**Keywords:** quality of life, mindfulness, meditation, case-control, plasma telomerase, healthy aging

## Abstract

Meditation involves psychophysical training which can result in a range of benefits including creating a calm mind and increasing self-awareness, relaxation, and tranquility. Increasing evidence, mostly based on short-term focused interventions, suggests that meditation-based activities may also have favorable effects on physical wellbeing including cellular aging. Hence, the aim of this study was to investigate if continued practice of meditation benefited quality of life, state of mindfulness, and plasma telomerase level in healthy adults. 30 long-term and skilled meditators were recruited from meditation centers in different parts of the island following a two-tier screening process of 70 eligible participants and 30 age- and gender-matched healthy non-meditators were recruited from the community. Mindfulness level and the quality of life were measured using the Five Facet Mindfulness Questionnaire (FFMQ) and Quality of Life Questionnaire, respectively, while the levels of plasma telomerase enzyme were measured using Enzyme-Linked Immunosorbent Assay. Skilled meditators had a better mindfulness level (*p* < 0.001) and quality of life (QOL; *p* < 0.001) than those in the comparison group. Similarly, higher plasma telomerase levels were observed in skilled meditators compared to non-meditators (*p* = 0.002). Trait mindfulness level and plasma telomerase level showed a significant relationship with the duration of meditation practice (*p* = 0.046 and *p* = 0.011, respectively). Regression analysis indicated that trait mindfulness level (*p* < 0.001) significantly predicts the plasma telomerase level. The findings of this comparative study add to the evidence on sustained benefits of meditation on wellbeing and healthy aging and supports incorporating meditation-based activities into lifestyle practices.

## Introduction

The fast paced and complex lifestyles with multiple demands at work and in personal lives lead to anxiety and stress experienced by many individuals in the present day. If the stress is acute, the human body typically recovers rapidly from it. However, ongoing chronic stress can cause or aggravate both mental and physical health problems including premature death (Rohleder, 2019). Although numerous psychological interventions have been introduced to alleviate these issues, there has been an exceptional focus on meditation-based interventions in the recent years. Meditation is a rigorous, spiritual, and psychophysical training that creates a connection between mind and body and it aids to achieve a clear, calm, and stable mind, self-awareness, relaxation, and tranquility (Cahn and Polich, 2006). Meditation has been perpetuated to this day in a variety of techniques including loving-kindness, body scanning, Zen, mindfulness, breathing, and concentration meditation which have its roots in different religions and traditions (Matko et al., 2021). Consistent with previous work describing the impact of positive psychological health on human longevity, meditation has gradually become a focus of scientific interest to promote healthy aging (Tolahunase et al., 2017). Moreover, quality of life (QOL), psychological attributes such as mindfulness and molecular processes including telomere maintenance related to aging are sensitive to meditative practices (Astin, 1997; Hoge et al., 2013; Alda et al., 2016; Chang et al., 2018).

Aging is a series of routes headed for death where each instant from birth influences the QOL, including quality of death. QOL is the ability to regulate and retain control of one’s own choices, feelings, behavior, healthiness, comfort, and bliss experienced by an individual (Luders et al., 2009; Tang et al., 2012). An increase in life expectancy and an aging population in many countries has renewed interest in approaches to improve and preserve the QOL of a person, which is intricately linked to ensuring good psychological and physical health (U.S. Department of Health and Human Services, 2021).

Mindfulness is a psychological process which is an inherent aspect of consciousness. It can be simply defined as one’s natural ability to pay and sustain attention to present-moment occurrences and it reflects individual differences in the general level of mindfulness across situations and time (Ludwig and Kabat-zinn, 2014). Even though initially advocated for cognitive benefits in older adults, mindfulness has shown to be equally beneficial in other age groups in society (Vickery and Dorjee, 2016; Fountain-Zaragoza and Prakash, 2017; Racey et al., 2018). Telomerase has been postulated as a psycho-biomarker which is used as an ultimate predictor of human age (Epel et al., 2009). It is a ribonucleoprotein enzyme complex which counteracts the replication-related attrition of telomeres, the protective end-caps of chromosomes (Mattern et al., 2004). Telomere length is recognized to affect the pace of aging and onset of age-associated diseases. Similarly reduced telomerase activity is associated with accelerated cellular senescence (Blackburn et al., 2015).

Recent preliminary investigations suggest that meditation can improve the mindfulness level and the QOL (Epel et al., 2009; Jacobs et al., 2010). Some studies have also reported that these outcomes may vary based on the form of meditation (Sharma, 2015; Le Nguyen et al., 2019; Puhlmann et al., 2019). Furthermore, telomerase activity has also been found to increase in mediation practitioners (Schutte and Malouff, 2014). However, the specific biological and psychological mechanisms which underpin the improved health outcomes brought about by meditation are still under explored.

**Figure 1:**
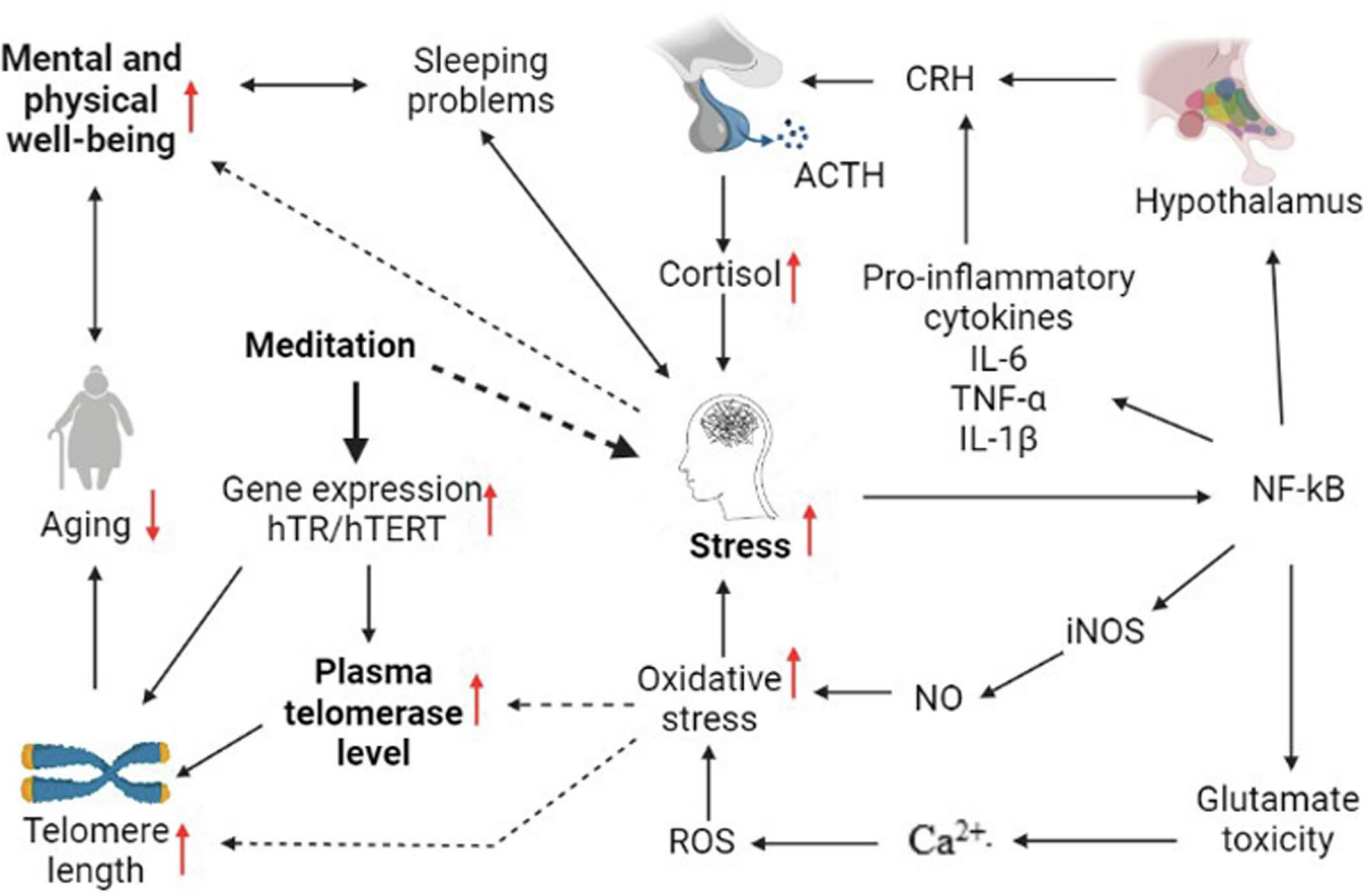
Schematic diagram of hypothesized biological pathway of the effects of meditation on mental and physical wellbeing and aging. NF-kB-Nuclear Factor kappa B; CRH-Corticotrophin-Releasing Hormone; ACTH-Adrenocorticotrophic Hormone; IL-6-Interleukin-6; TNF-α-Tumor Necrosis Factor-alpha; IL-1β-Interleukin-1 Beta; Ca^2+^-Caylcium; ROS-Reactive Oxygen Species; iNOS-inducible Nitric Oxide Synthase; NO-Nitric Oxide; and ---> Inhibitory effect. The production of ROS and iNOS which is triggered by an internal/external stressor, increases oxidative stress, which in turn reduces plasma telomerase enzyme level and telomere length (Saretzki, 2009; Fouquerel et al., 2016). Decreased telomere length is associated with rapid aging (Vaiserman and Krasnienkov, 2021). The Hypothalamic Pituitary Adrenal (HPA) axis is activated when the brain perceives a stressor and as a result cortisol is released. Increased cortisol and oxidative stress cause physical and mental stress (Kasala et al., 2014). Stress also has a negative effect on mental and physical wellbeing (Rector et al., 2016). Meditation can improve wellbeing and mindfulness by reducing stress and thus, reduce aging (Chételat et al., 2018). Meditation promotes the expression of the hTERT gene, which encodes for a component of telomerase enzyme. Plasma telomerase acts on the telomeres and maintains its length. Meditation also promotes the expression of hTR gene which provides a template for the production of the repeated sequence tracts on telomeres (Buric, 2017) and reduces the shortening of telomere lengths which in turn delays cellular aging (Figure created with BioRender.com).

While these positive effects have been mostly demonstrated singularly in independent populations, exploring multiple attributes and outcomes in the same study group would lead to a better understanding of their inter-relatedness and how they could be harnessed for betterment of the wider population. Therefore, this study aimed to investigate the effect of long-term meditation on the QOL, mindfulness level, and levels of plasma telomerase enzyme in healthy individuals. The hypothesized schematic diagram of the biological effects of meditation leading to positive psychological and physical outcomes is outlined in Figure 1. The decision to focus on a general population was motivated by the fact that although the clinical benefits of meditation are more appealing, a part of the academic world has begun to examine the effects of meditation on non-clinical populations with notable and promising results.

## Materials and Methods

### Participants and Setting

To be included in the study, the participants had to be aged between 18 and 65 years of age. Meditators should have attended at least 1 week of continuous retreats or a temple-based meditation session and must have meditated at least 6 hours per week during the preceding 3 years. Age (±2 years), gender, and educational level matched controls (non-meditators) were selected if they had never or rarely (less than once in 3 months) practiced meditation, yoga, or mindfulness practice. The criteria for exclusion included as: (i) individuals who had history or current diagnosis of a psychiatric disorder (based on the responses to questions) and (ii) individuals who were receiving medication or psychological treatment or were suffering from any illness that could affect plasma telomerase level (such as any type of cancer or Acquired Immune Deficiency Syndrome).

**Figure 2:**
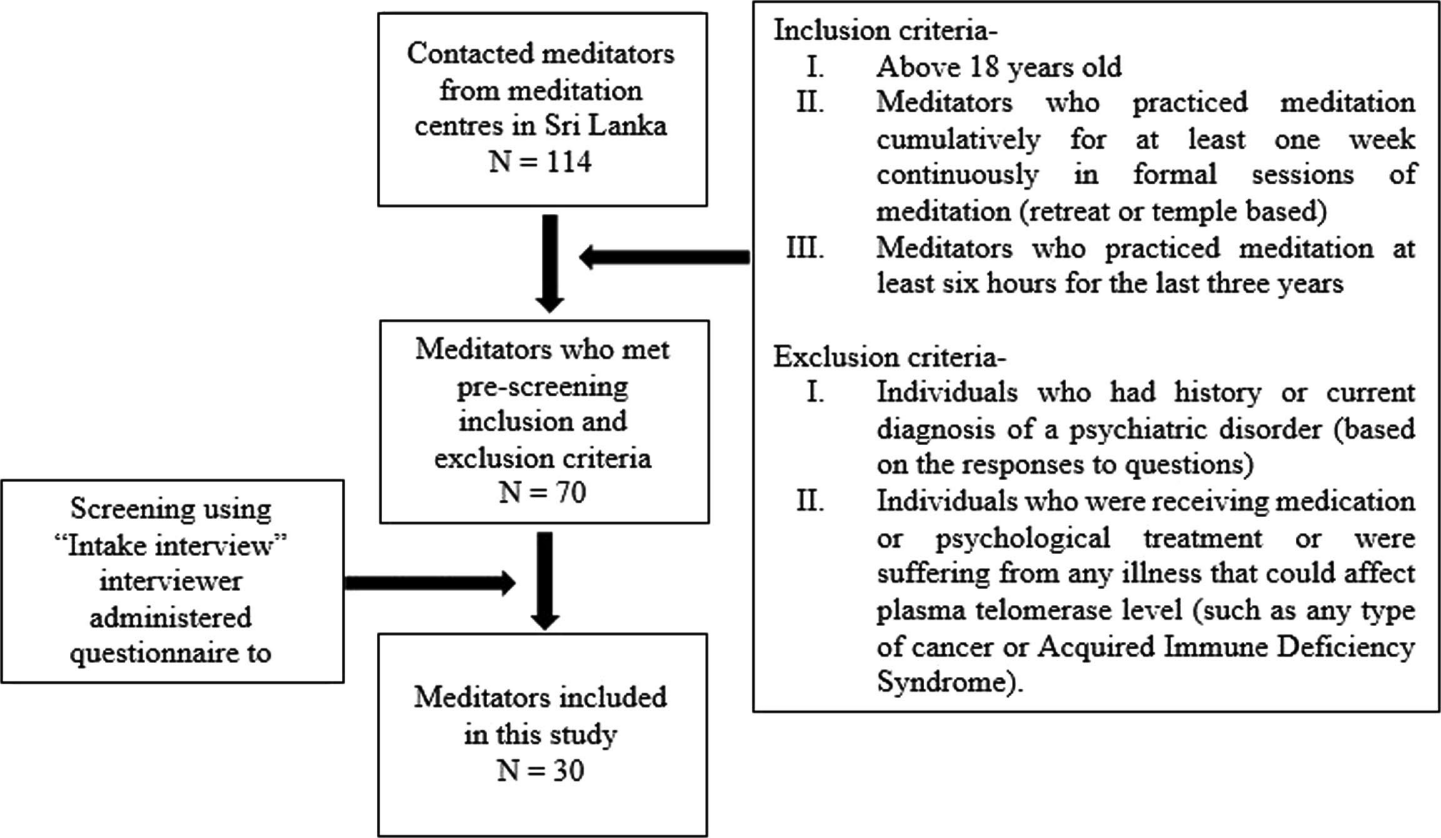
The process of recruiting skilled meditators to the study.

Initially, 114 meditators were contacted from meditation centers in Colombo, Mathale, Kandy Kalutara, Gampaha, Kurunegala, and Galle districts in Sri Lanka and 70 of the meditators fulfilled the inclusion and exclusion criteria. Even though they fulfilled the eligibility criteria, they were further screened to identify skilled meditators using a questionnaire-based screening tool which was developed and validated as a part of this study through the following steps: item generation through critical literature search and interviewing expert meditators, focus group discussions, Delphi group discussions, translations and back translations, pre-testing and pilot testing with expert meditators. The screening tool which consisted of 30 questions analyzed six features: duration of meditation practice, details of meditation practice, stable attention, heightened peripheral awareness, alertness, and emotional stability. The meditators who had more than the ideal total recruitment score, i.e., 72 out of 80, were identified as skilled meditators (the ideal recruitment scores are intended for each section; Figure 2).

The total sample size for the study was calculated to be 60 participants (30 each for meditators and controls) using G Power Software (Kang, 2021) to provide an effect size of 0.8 and 80% power for an alpha error of 0.05. 30 meditators who met the skilled meditator criteria according to the predetermined scores were recruited into the study. 30 controls were recruited from the community using purposive sampling to match for the meditators. Participants provided informed written consent to take part in the study. Ethics approval was granted by the Ethics Review Committee, Faculty of Medicine, University of Colombo, Sri Lanka (EC-19-067) and recruitment was conducted between August 15 and October 15, 2021.

### Design

This study is a case-control study where the cases (meditators) were matched with controls (non-meditators) for age (±2 years), gender, and highest educational level. The cases were matched one to one with a non-meditator control.

### Assessment

#### Physical and Mental Health Measurement

Three questionnaires were used to assess (i) QOL, (ii) Mindfulness level, and (iii) Socio-demographic features.

##### Quality of Life

QOL was measured using the abbreviated version of the World Health Organization Quality of Life Questionnaire (WHOQOL-BREF; Kumarapeli et al., 2006). WHOQOL-BRFE scale has 26 items, divided into four domains of QOL, i.e., physical health, psychological health, social relationships, and environment. Answers to all the questions are rated on a Likert scale with five options: absolutely not (scored as “1”), slightly (“2”), quite a bit (“3”), most likely (“4”), and very well (“5”). The participants chose the most appropriate option that describes the intensity of their feelings at the time of answering.

##### Mindfulness Level

Mindfulness level of the individuals was measured using Five Facet Mindfulness Questionnaire (FFMQ; Baer et al., 2008) which was judgmentally validated into Sinhala language by one of the research team members. FFMQ consists of 39 items and it assesses five facets of mindfulness which focuses on the following five elements of mindfulness: observing (noticing or attending to internal and external experiences, such as sensations, cognitions, emotions, sights, sounds, and smells), describing (labeling internal experiences with words), acting with awareness (focusing on one’s activities at a given moment), non-judging of inner experience (taking a non-evaluating stance toward thoughts and feelings), and non-reactivity to inner experience (tendency to allow thoughts and feelings to come and go, without being carried away by or caught up in them). These items are rated according to the Likert scale: never (scored as “1”), rarely (“2”), sometimes (“3”), often (“4”), and very often (“5”).

##### Socio-Demographic Data

Age, gender, educational level, marital status, body mass index (BMI), sleeping hours, working hours, and lifestyle habits including alcohol consumption, dietary patterns, and period of physical exercise (hours) were collected through an interviewer administered questionnaire.

The above-mentioned scales were administered among both long-term meditators and control groups. Participants were allowed to complete the questionnaires (QOL and FFMQ) at their own place with no time limitations imposed for completion in order to make sure each item of each questionnaire was carefully considered and to ensure less participant fatigue. Participants were also given the option to ask questions during the completion of questionnaires.

#### Blood Collection

Participants arrived at the laboratory between 8 am and 9 am. Peripheral blood was drawn from the participants *via* venepuncture using vacutainers into tubes containing Ethylenediamine tetraacetic acid (EDTA) for plasma separation *via* centrifugation.

#### Plasma Telomerase Level Measurement

The level of plasma telomerase was measured using a commercially available Human TE (Telomerase) ELISA assay kit (Elabscience, United States) according to the manufacturer’s instructions. The test uses a quantitative sandwich enzyme-linked immunosorbent assay where the detection limits for plasma telomerase enzyme range between 0 and 10 ng/ml. Twofold dilution series ranging from 0 to 10 ng/ml were prepared using 10 ng/ml of recombinant human telomerase stock solution in order to gain a standard curve. Assay diluent solution provided in the kit was used for the preparation of standards. Samples were tested in duplicate and the optical density (OD value) of each well was read at 450 nm. The concentrations of the plasma telomerase level were interpolated using the standard curve and the mean values of the samples were considered for analysis.

### Statistical Analysis

Data were summarized and presented using appropriate descriptive statistics. Normality of the data was measured using Shapiro Wilk test. Comparison of the two study groups for homogeneity in baseline characteristics and lifestyle habits was conducted using Chi-square test for categorical variables and Independent sample *t*-test for continuous variables. Independent sample *t*-test was used to compare QOL, mindfulness level, and plasma telomerase concentration between the two groups. Relationships between trait mindfulness level, QOL, and plasma telomerase concentration with socio-demographic factors were assessed using Pearson correlation. Multiple regression analysis was used to forecast if trait mindfulness level and QOL significantly predicted the plasma telomerase levels. All statistical analyses were carried out using IBM SPSS (Version 23.0), and values of *p* < 0.05 were considered significant.

## Results

The demographic and health characteristics of both cases and controls are outlined in Table 1. 19 of the 30 participants (63.34%) in each group were male. The average age (±SD) of participants was 43.83 ± 9.92 years and 43.51 ± 9.92 years for the meditators and non-meditators, respectively. All the participants were Sinhalese, and their religion was Theravada Buddhism. Mean duration of the meditation practice of the meditators was 6.80 ± 3.27 years and they had meditated for a mean period of 5.82 ± 3.45 h per day. Meditators had spent a mean cumulative number of 47 days in retreats within the previous year. Loving-kindness, breathing, and body scanning were the meditation techniques practiced by the meditators included in the study. The comparison of baseline characteristics and lifestyle habits showed no significant differences between the two groups.

**Table 1:** Socio-demographic and health characteristics of the study sample.

Variables	Meditators	Non-meditators	Significance
*Socio-demographics*		
Gender (male)^a^	19/30(63.34%)	19/30(63.34%)	*p* = 1
Age (mean, SD)^a^	43.83 ± 9.92	43.51 ± 9.92	*p* = 0.897
Married (%)	19/30(63.34%)	14/30(46.6%)	*p* = 0.407
Educational level^a^- Tertiary education	24/30(80%)	24/30(80%)	*p* = 1
Educational level^a^- Secondary education	6/30(20%)	6/30(20%)	*p* = 1
Body Mass Index (mean, SD)	26.5 ± 5.23	23.39 ± 2.61	*p* = 0.227
*Lifestyle and habits*		
Alcohol^b^ (%)	7/30(23.3%)	8/30(26.67%)	*p* = 1
Smokers (%)	0/30(0%)	0/30(0%)	*p* = 1
Non-vegetarian diet	29/30(96.67%)	30/30(100%)	*p* = 1
Sleeping hours per day	6.27 ± 1.56	6.22 ± 1.92	*p* = 0.987
Exercise (>1 h/week)	8	13	*p* = 0.1
	8/30(26.67%)	13/30(43.34%)	*p* = 0.601

a *Matched variables*.

b *Consume alcohol occasionally*.

Table 2 summarizes the comparison of indicators of QOL and mindfulness between the meditators and non-meditators. The expert meditators had a significantly higher trait mindfulness level (mean ± SD, meditators: 156.67 ± 19.54, controls: 126.68 ± 18.47, *p* < 0.001) and higher overall QOL (mean ± SD, meditators: 72.78 ± 11.07, controls: 57.34 ± 7.11, *p* < 0.001).

**Table 2:** Mindfulness, wellbeing, and plasma telomerase concentration in mediators and non-meditators.

Variable	Meditators (mean ± SD)	Non-meditators (mean ± SD)	Significance
*Facets of mindfulness* FFMQ (Total)	156.67 ± 19.54	126.68 ± 18.47	*p* < 0.001^*^
FFMQ (Observing)^a^	30.04 ± 5.30	24.51 ± 4.52	*p* < 0.001^*^
FFMQ (Describing)^b^	32.83 ± 3.9	26.31 ± 3.91	*p* < 0.001^*^
FFMQ (Acting aware)^c^	34.32 ± 5.04	25.37 ± 4.68	*p* < 0.001^*^
FFMQ (Non-judging)^d^	30.32 ± 5.90	21.25 ± 3.15	*p* < 0.001^*^
FFMQ (Non-reacting)^e^	29.03 ± 4.18	23.09 ± 3.08	*p* < 0.001^*^
*Quality of life* QOL (Total)	72.78 ± 11.07	57.34 ± 7.11	*p* < 0.001^*^
QOL (Physical health)	15.07 ± 1.24	14.04 ± 1.76	*p* = 0.012^*^
QOL (Psychological)	16.39 ± 1.14	12.64 ± 2.64	*p* < 0.001^*^
QOL (Social Relationship)	16.09 ± 2.67	14.51 ± 3.81	*p* < 0.001^*^
QOL (Environment)	17.24 ± 2.02	14.40 ± 1.98	*p* < 0.001^*^
Plasma telomerase level (ng/ml)	8.82 ± 2.51	6.42 ± 3.24	*p* = 0.002^*^

*SD, Standard deviation; FFMQ, Five facet mindfulness questionnaire; and QOL, Quality of life*.

* *p < 0.05*.

a *The way a person uses his sensory organs, such as how he sees, feels, and perceives the internal and external environment around him*.

b *The way a person labels his experiences and expresses them in words to himself and others*.

c *The movements a person chooses after attending to the information present at the moment*.

d *This is call for self-acceptance and unconditional empathy for oneself and others*.

e *How a person actively detaches from negative thoughts and emotions to accept their existence and choose not to react to them*.

Each facet of the mindfulness questionnaire and each domain of the QOL questionnaire were measured and analyzed separately and showed a similar trend to overall measures. Accordingly, the mean scores of all the mindfulness subscales including observing (mean ± SD, meditators: 30.04 ± 5.30, controls: 24.51 ± 4.52, *p* < 0.001), describing (mean ± SD, meditators: 32.83 ± 3.9, controls: 26.31 ± 3.91, *p* < 0.001), acting with awareness (mean ± SD, meditator: 34.32 ± 5.04, controls: 25.37 ± 4.68, *p* < 0.001), non-judging (mean ± SD, meditators: 30.32 ± 5.90, controls: 21.25 ± 3.15, *p* < 0.001), and non-reacting (mean ± SD, meditators: 29.03 ± 4.18, controls: 23.09 ± 3.08, *p* < 0.001) were significantly higher in meditators compared to non-meditators. Scores of the four domains of QOL including physical health (mean ± SD, meditators: 15.07 ± 1.24, controls: 14.04 ± 1.76, *p* = 0.012), psychological health (mean ± SD, meditators: 16.39 ± 1.14, control: 12.64 ± 2.64, *p* < 0.001), Social relationship (mean ± SD, meditators: 16.09 ± 2.67, controls: 14.51 ± 3.81, *p* < 0.001), and Environment (mean ± SD, meditators: 17.24 ± 2.02, controls: 14.40 ± 1.98, *p* < 0.001) were significantly higher in meditators. Further, meditators had significantly higher plasma telomerase levels than non-meditators (mean ± SD, meditators: 8.82 ± 2.51 ng/ml, controls: 6.42 ± 3.24 ng/ml, *p* = 0.002).

Moreover, a significant negative correlation was observed between age and plasma telomerase level (*r* = −0.666, *p* < 0.001), whereas age did not show any significant relationship with QOL and trait mindfulness level. However, the other demographic features including gender, highest educational level, BMI, marital status, exercise hours per week, sleeping hours per day, number of hours spent outdoors per day, and maternal and paternal age at birth did not show a correlation with trait mindfulness level, QOL, or the plasma telomerase concentration (*p* > 0.05). Duration of the meditation practice of the long-term meditators also showed a significant relationship with the trait mindfulness level (*r* = 0.361, *p* = 0.046) and plasma telomerase level (*r* = 0.450, *p* = 0.011) but did not show a significant relationship with QOL (*r* = 0.044, *p* = 0.815). It was found that trait mindfulness level (*R*^2^ = 0.647, *p* < 0.001) significantly predicts the plasma telomerase level when considering the regression analysis.

## Discussion

In the present study, we examined healthy, long-term meditators for multiple biomarkers of positive psychological and physical health and compared them against a group of matched meditation-naïve individuals. We observed that meditators had significantly higher levels of mindfulness, QOL, and plasma telomerase compared to age- and gender-matched non-meditators. Our results show that long-term meditators are more adept at identifying and labeling their experiences, self-acceptance, and detachment from negative thoughts and emotions. We also show that long-term meditators have higher a perception of their wellbeing, health, and life satisfaction as reflected by higher subscores across all key domains of QOL. The finding of higher mindfulness levels and QOL in meditators is consistent with previous work that demonstrated an association between meditation with mindfulness and QOL (Lilja et al., 2013; Chang et al., 2018). These results strengthen the impression that mental health and behavioral patterns are influenced by the manner in which people relate to their thoughts and feelings rather than by the form of those thoughts and feelings.

Telomerase is an enzyme and hence telomerase activity will be influenced by its concentration. The increased activity of telomerase in the meditators described in the published literature is in agreement with our results and supports assay of telomerase levels in peripheral blood as a surrogate marker of telomerase activity (Schutte and Malouff, 2014). Our results showed a significant association between trait mindfulness with plasma telomerase level and also that mindfulness and plasma telomerase improved with time spent on meditation. This indicates the possible inter-relationship between meditation and cellular aging, mindfulness, and QOL. Meditation improves mindfulness and QOL which may then lead to individuals experiencing less stress and anxiety. Low stress and anxiety are associated with higher levels of telomerase and telomere lengths (Cahn and Polich, 2006; Epel et al., 2009; Conklin et al., 2018; Epel and Prather, 2018) which may decrease cellular aging (Epel et al., 2009). Thus, the results of this study suggest that meditation could potentially have beneficial effects on the plasma telomerase level which is linked to longevity and delaying of the cellular aging. Aging is well recognized to reduce mental and physical wellbeing (Chételat et al., 2018). Therefore, in addition to its direct effects on psychological health, meditation will improve mental and physical wellbeing through its positive effects on aging.

We recruited meditation practitioners who practiced meditation for more than 3 years to seek the long-term effects of meditation and a screening tool was used to properly identify the meditators who gained the necessary skill levels, which would have contributed to the significant differences observed even with a limited sample size. A higher percentage (63.34%) of meditators were males and this may be partly due to sex differences in motivation to practice meditation or/and to participate in research studies. According to the educational levels, most of the meditators had completed their tertiary education which indicates that most of the higher educated people tend to practice meditation and achieve the skill levels. Efforts were taken to avoid bias in participant recruitment by administering the questionnaire to a larger group of meditators to select the skilled meditators. All the meditators who scored more than the ideal score participated in this study. Further, the controls were recruited from the community using purposive sampling where all the contacted controls also participated in the study. Previous studies have shown similar results for the practice of mindfulness meditation (Schutte and Malouff, 2014; Chang et al., 2018), loving-kindness meditation (Hoge et al., 2013; Le Nguyen et al., 2019), and Zen meditation (Alda et al., 2016). However, all the meditation techniques were considered as one technique in our study because it is known that meditation has beneficial effects regardless of the type of meditation practice. While many studies have reported benefits of meditation, evaluated after a short-term structured program, our findings strengthen and add to the evidence for more sustained effects of meditation practices (Lykins and Baer, 2009; Hoge et al., 2013; Alda et al., 2016; Mendioroz et al., 2020). Since we focused on a healthy population, the results are more applicable to a wider population and the findings of this study could serve as a baseline to study the effects of meditation in a clinical population.

This study has a number of limitations that warrants a cautious explanation of the results. Simultaneous measuring of telomerase activity and the length of the telomeres will provide a stronger and more valid conclusion of the plasma telomerase levels and its predictive value of cellular aging. While the results suggest positive influences with all techniques adopted by participants in this study, which would be an advantage in promoting meditative practices of one’s preference, the findings should be confirmed by separately comparing all three techniques: loving-kindness, breathing, and body scanning meditation with controls and with each other. A sub-analysis based on age groups would provide a more precise picture of the dynamics of telomerase which was not attempted due to the limited sample number. However, the meditators were closely matched (±2 years) with non-meditators to minimize the effects due to variability in age. This study did not measure markers of stress such as serum cortisol levels in the participants or other markers of inflammation and general redox status which are recognized to affect telomerase activity. Nonetheless, the meditator cohort and sample repository established during this study offers an intriguing line of future research to explore the dynamics of multiple psychological, physical, and biochemical parameters which may affect wellbeing and cellular aging.

## Conclusion

The results of this case-control study suggest that long-term meditators experience enhanced psychosocial wellbeing, such as a better outlook on life experiences and coping skills, a better physical QOL as well as underlying biochemical changes that would contribute to healthy aging. Thus, this study adds to the evidence on sustained benefits of meditation and supports incorporating meditation-based activities into lifestyle practices.

## Data Availability Statement

The raw data supporting the conclusions of this article will be made available by the authors, without undue reservation.

## Ethics Statement

The studies involving human participants were reviewed and approved by the Ethics Review Committee, Faculty of Medicine, University of Colombo, Sri Lanka. The patients/participants provided their written informed consent to participate in this study.

## Author Contributions

The authors confirm contribution to the paper as follows. NS and NSi: study conception and design. ND: data collection, analysis, and interpretation of results. ND, NSi, and NSa: draft manuscript preparation. All authors contributed to the article and approved the submitted version.

## Funding

This work was supported by a grant from the Accelerating Higher Education Expansion and Development (AHEAD) Operation of the Ministry of Higher Education funded by the World Bank (grant no. 6026-LK/8743-LK).

## Conflict of Interest

The authors declare that the research was conducted in the absence of any commercial or financial relationships that could be construed as a potential conflict of interest.

## Publisher’s Note

All claims expressed in this article are solely those of the authors and do not necessarily represent those of their affiliated organizations, or those of the publisher, the editors and the reviewers. Any product that may be evaluated in this article, or claim that may be made by its manufacturer, is not guaranteed or endorsed by the publisher.

## Abbreviations

BMI: Body Mass Index
ELISA: Enzyme-Linked Immunosorbent Assay
FFMQ: Five Facet Mindfulness Questionnaire
SD: Standard Deviation
QOL: Quality of life
WHOQOL-BRFE: World Health Organization Quality of Life Instruments
